# Immunological Characteristics of Alternative Splicing Profiles Related to Prognosis in Bladder Cancer

**DOI:** 10.3389/fimmu.2022.911902

**Published:** 2022-06-13

**Authors:** Fangdie Ye, Yingchun Liang, Zhang Cheng, Yufei Liu, Jimeng Hu, Weijian Li, Xinan Chen, Jiahao Gao, Haowen Jiang

**Affiliations:** ^1^ Department of Urology, Huashan Hospital, Fudan University, Shanghai, China; ^2^ Fudan Institute of Urology, Huashan Hospital, Fudan University, Shanghai, China; ^3^ Department of Radiology, Huashan Hospital, Fudan University, Shanghai, China; ^4^ National Clinical Research Center for Aging and Medicine, Huashan Hospital, Fudan University, Shanghai, China

**Keywords:** tumor microenvironment, immunotherapy targets, prognostic, bladder cancer, alternative splicing

## Abstract

Several studies have found that pathological imbalance of alterative splicing (AS) events is associated with cancer susceptibility. carcinogenicity. Nevertheless, the relationship between heritable variation in AS events and carcinogenicity has not been extensively explored. Here, we downloaded AS event signatures, transcriptome profiles, and matched clinical information from The Cancer Genome Atlas (TCGA) database, identified the prognostic AS-related events *via* conducting the univariate Cox regression algorism. Subsequently, the prognostic AS-related events were further reduced by the least absolute shrinkage and selection operator (LASSO) logistic regression model, and employed for constructing the risk model. Single-sample (ssGSEA), ESTIMATE, and the CIBERSORT algorithms were conducted to evaluate tumor microenvironment status. CCK8, cell culture scratch, transwell invasion assays and flow cytometry were conducted to confirm the reliability of the model. We found 2751 prognostic-related AS events, and constructed a risk model with seven prognostic-related AS events. Compared with high-risk score patients, the overall survival rate of the patients with low-risk score was remarkably longer. Besides, we further found that risk score was also closely related to alterations in immune cell infiltration and immunotherapeutic molecules, indicating its potential as an observation of immune infiltration and clinical response to immunotherapy. In addition, the downstream target gene (DYM) could be a promising prognostic factor for bladder cancer. Our investigation provided an indispensable reference for ulteriorly exploring the role of AS events in the tumor microenvironment and immunotherapy efficiency, and rendered personalized prognosis monitoring for bladder cancer.

## Introduction

Bladder carcinoma is a common type of genitourinary system tumor worldwide, and carries a significant burden, responsible for an estimated 570000 new cases and 210 000 deaths annually (1). As a heterogeneous tumor, bladder carcinoma mainly progresses along two “trajectories”, and each “trajectory” has distinct effects on its for prognosis. The one “trajectories” was non-muscle invasive bladder cancer which was recurrent noninvasive tumors managed chronically, while muscle-invasive bladder cancers are progressive-stage or aggressive diseases that require multi-strategy treatment (2). Although remarkable breakthroughs in investigating the underlying biological mechanisms of bladder cancer have basically improved the diagnosis and treatment of this disease, the histology of bladder cancer is highly variable, potentially representing different molecular subtypes, which adds to the complexity of management (2, 3). Increasing number of articles have elaborated that genetic subtype may associated with distinct clinical responses to biotherapies, chemotherapies, and survival outcomes, confirming their clinical relevance (4–6). However, owing to high levels of inter-observer variability, judging these subtypes may be subjective, leading the human-bias for diagnosis, therapeutic benefits, and prognosis. Thus, there is imperative to understand the underlying mechanisms of genetic subtypes from multi-aspects and identify predictable biomarkers.

The tumor immune microenvironment (TIME) includes an array of immunocytes, including macrophages, T cells, neutrophiles, DC cells and NK cells. Increasing research demonstrated that the immunocytes in the tumor microenvironment interact with therapeutic drug, thereby affecting the clinical response of patients to treatment. These immunocytes could therefore act as targets to promote the overall survival of patients with bladder cancer (7–9). Recently, immunotherapy has yielded encouraging results in numerous malignancies and has received extensive attention (10). Bladder cancer has also been successfully treated by several immunotherapeutic strategies, such as Bacillus Calmette–Guerin (BCG) intravesical instillation or PD‐L1antibody treatment. Nevertheless, the mechanisms of BCG‐induced tumor-specific immunity remain obscure, and only 25% of progressive bladder cancers have remarkable clinical respond to immunotherapy treatment (11, 12). Therefore, the most effective strategy for accurately predicting the response of bladder cancer to immunotherapy or cancer progression may be based on the strategy of molecular risk distribution, which can help identify bladder cancer patients online through specific molecular characteristics, improve the prognosis accuracy, and optimize the benefit of immunotherapy.

Exact gene is incised *via* Alternative splicing (AS) to yield a quantity of special messenger RNA (mRNA) (13). It is known that AS includes seven types: mutually exclusive exons (ME), exon skip (ES), alternate terminator (AT), alternate promoter (AP), retained intron (RI), alternate donor site (AD), and alternate acceptor site (AA) (14). During tumor development, AS process changes abnormally, and alterations in critical tumor genes can play a pivotal role in oncogenesis, tumor progression, metastasis, and therapeutic response (15–18). Besides, some splicing factors have been confirmed to play a crucial role in the regulation of AS events (19). Notably, anomalous alternation of pivotal splicing factors can lead to the formation of carcinogenic splicing isoforms (20–22). heretofore, several studies have focused on exploring the function of AS-related mutation in bladder cancer (23–25).Recently, some articles have focused on the AS-based prognostic model of bladder cancer (26, 27). However, the correlation between prognostic-related AS events and immunotherapy/TIME is still unclear. Therefore, we conduct in-depth investigation of aberrant AS events to demonstrate the profiles of tumor microenvironment and the potential biological mechanisms of oncogenesis, further optimizing diagnosis, prognosis, and clinical strategies.

In our research, we outlined the AS-pattern and ascertain that AS events were closely related to the TIME and clinical outcome *via* comprehensive bioinformatic analysis based the TCGA-BLCA cohort. Next, we revealed downstream target genes (DYM) for prognostic-related AS events. The latent role of DYM in bladder cancer has also been explored. At the same time, we confirmed that DYM is associated with alteration of TIME, and silencing DYM can inhibit the cell proliferation, migration, invasion ability, and promote cell apoptosis.

## Material and Methods

### Acquisition of Multi-Omics Data Related to Bladder Cancer

In the and identify Cancer Genome Atlas (TCGA) SpliceSeq database, the alternative splicing events, including ME, ES, AT, AP, RI, AD, AA, were analyzed and summarized using the R package “Upset.” The characteristics of AS events were interpreted using the percent spliced in (PSI), which is an index that can qualify variable splicing. AS event annotation: gene symbol, splicing type and splicing ID number. The transcriptome FPKM information and adjusted clinical data were acquired from the TCGA database. Patients’ selection criteria: pathological result was transitional cell papilloma and carcinoma. Exclusion criteria: 1. Patients with less than 10 days of survival. 2. Patients without corresponding alternative splicing data. A total of 409 patients diagnosed with transitional cell papilloma and carcinoma, 13 patients were excluded by exclusion criteria, 396 patients were left. Deleting patients with missing clinical features when performing correlation analysis among risk score and clinicopathological profiles. The clinical data on patient’s immunotherapy were collected from TCIA(https://www.tcia.at/home).

### Construction and Validation of AS Events-Related Prognostic Signatures

The clinical information and corresponding AS events of the samples were matched according to the splicing ID number. Then, the prognostic-related AS events were identified *via* conducting univariate Cox regression algorism, which are displayed as a volcano map and Upset diagram. In addition, the top 20 AS events are presented in the quadrangle plot.

To construct a valuable prognostic model, LASSO regression analysis was employed to lessen the dimension of prognostic-related AS events and to select candidate features with prominent prognostic value. On this basis, multivariate Cox model was conducted to determine the final prognostic-related AS events, which were utilized to propose the prognostic model in this study. The formula was calculated as follows:


Risk score=coefficient 1 × PSI AS event 1+coefficient 2 × PSI AS event 2 + ⋯ +coefficient n ×PSI AS event n.

The patients were divided into high- and low-risk subgroups by determining the median risk score. Then the K-M survival curve was portrayed to estimate the difference of clinical outcome between two subgroups. Besides, the receiver operating characteristic (ROC) model depicts the clinical predictive performance of two subgroups. The forest was plotted to determine whether the risk score can independently predict the clinical outcome of patients.

To comprehensively assess the prognosis of each patient with bladder cancer, nomogram model which included the risk score, tumor stage, age, gender, and WHO grade was constructed. Subsequently, the calibration curve was calculated to evaluate the 1-, 3-, 5- year overall survival probabilities.

### Characteristic of the Immune Microenvironment

To investigate the infiltration situation of immunocytes in the tumor microenvironment, three classical analyses were performed in this study. (1) The single sample gene-set enrichment analysis (ssGSEA) was conducted to explore the proportion of 29 immunocyte types in two distinct risk subgroups according to the previous publication (the gene-set was show in **Table S1**) (28). (2) The R package “ESTIMATE” was executed to evaluate the immune/stromal cell infiltration, which could indict the difference of TME between two distinct risky subgroups. (3) R package “CIBERSORT” was conducted to examine the proportion of 22 immunocyte types for each sample (the gene-set was show in **Table S2**) (29).

### Effect of AS Events on ICB Treatment

Recent studies have indicated that the transcriptome of ICB-related genes may be closely related to clinical response of patients to immunotherapy. In this study, 47 ICB-related genes were extracted, such as programmed death 1 (*PD‐1, also named PDCD1*), programmed death ligand 1 (*PD‐L1*/*CD274*), the more information ICB-related genes were seen in **Table S3** (30). The Spearman correlation algorism was conducted to calculated the association between ICB-related genes and risk score to speculate the effect of immunotherapy.

### Cell Culture and Infection

T24 and J82 bladder cancer cells were gained from the Type Culture Collection of the Chinese Academy of Sciences (Shanghai, China). The cells were maintained in DMEM medium with 10% fetal bovine serum at 37°C in cell incubator with 5% CO_2_. 3 × 10^5^ bladder cancer cells line were seeded into 6-well dishes, cultured for 24h, then transfected by using Lipofectamine 3000. biological experiments were carried out according to the appropriate transfection time. DYM transfection was identified using quantitative real-time PCR (qRT-PCR). The si-RNA sequences were listed as following: si-DYM-1: 5′-GGGUCCUGGAAAUCAUUAATT-3′, si-DYM-2: 5′-GGAGGAAGCAACCAUUUCATT -3′, si-con: 5′-UUCUGGCAACGUATCAGCUTT-3′.

### Macrophage Polarization

THP-1 cells were donated by Dr. Cai from Shanghai Jiaotong University. THP-1 cells were induced to differentiate into M0 macrophages by 100 ng/ml PMA. In order to simulate the formation of tumor-associated macrophages(TAMs), the Falcon^®^ Cell Culture Inserts (Corning, Corning, NY) was employed to construct the co-culture environment, the bladder cancer cells (T24/J82) were inoculated in the upper chamber, and M0 macrophages were inoculated in the lower chamber to achieve the effect of co-culture. After 48 hours, co-cultured macrophages were collected to obtain TAMs. CD206 and CD163 were used as markers of M2-type macrophages, and CD86 as markers of M1-type macrophages

### ELISA

ELISA kit (R & D Systems) was used to detect the levels of IL6, IL-10, CCL2 and CCL3 in supernatant. The average values of the three independent experiments were shown by the histogram.

### RNA Isolation and qRT-PCR

In order to verify knockdown efficiency, we extracted the purity RNA from cell lines *via* TRIzol Reagent (Invitrogen), and then SuperScript II Reverse Transcriptase (Invitrogen) was employed to transcribed mRNA into. The qRT-PCR reaction was conducted using an AB7300 thermocycler (Applied Biosystems). The relative expression of cDNA was normalized to that of *GAPDH*, and each reaction contained at least three separate biological replicates. The primers used are listed in **Table S4**.

### Cell Proliferation Assay

For the cell counting kit-8 (CCK-8) assay, each experimental group was inoculated with a density of 2000/well in 96-well plates. After 1, 2, and 3 d, 110 µL mixed solution (CCK-8 + DMEM) was added to each 96-well plates and the cells were cultured for another 2 h. OD_450_ was measured to assess cell proliferation status.

### Cell Migration and Invasive Ability

Cell migration and invasive ability were evaluated by cell scratch assay and transwell invasion assay, respectively. For the cell culture scratch assay, 2 × 10^5^ BCa cells were seeded into six-well plates. After covering the whole plates, the cells were scratched with 1 mL pipette tips. The gap area was recorded at 0, 24, and 48 h, and assessed using Image J software. For the transwell invasive assay, 150 µL DMEM medium with 10% FBS was added into the lower chamber and 2 × 10^4^ cells were seeded into the upper chambers. After 24 h, removed the cells which located on the upper surface of the chamber, and stained the invading cells on the lower chamber *via* crystal violet. The invaded cells were photographed and calculated in three random fields.

### Flow-Cytometric Analysis

T24 and J82 cells were seeded into six-well plates, The cells were transfected with Si-con and Si-DYM for 24 hours in each well, then digested with trypsin and processed with cold PBS (4° C). We collected the suspension cells in the flow tube according to the manufacturer’ s protocol. Finally, apoptosis was measured by using BD FACS caliber. All experiments were conducted in triplicate.

### Statistical analysis

All data analyses were conducted *via* using the R software (version 4.0.2). The Wilcoxon test was carried out for comparative analysis of the two group characteristics, and the Kruskal-Wallis test was conducted for comparative analysis more than two group characteristics. Correlations between risk score, clinical characteristics, and other variables were calculated using the Pearson correlation test. The experiments were repeated at least three times.

## Results

### Identification of Prognostic-Related AS Events

**Figure 1:**
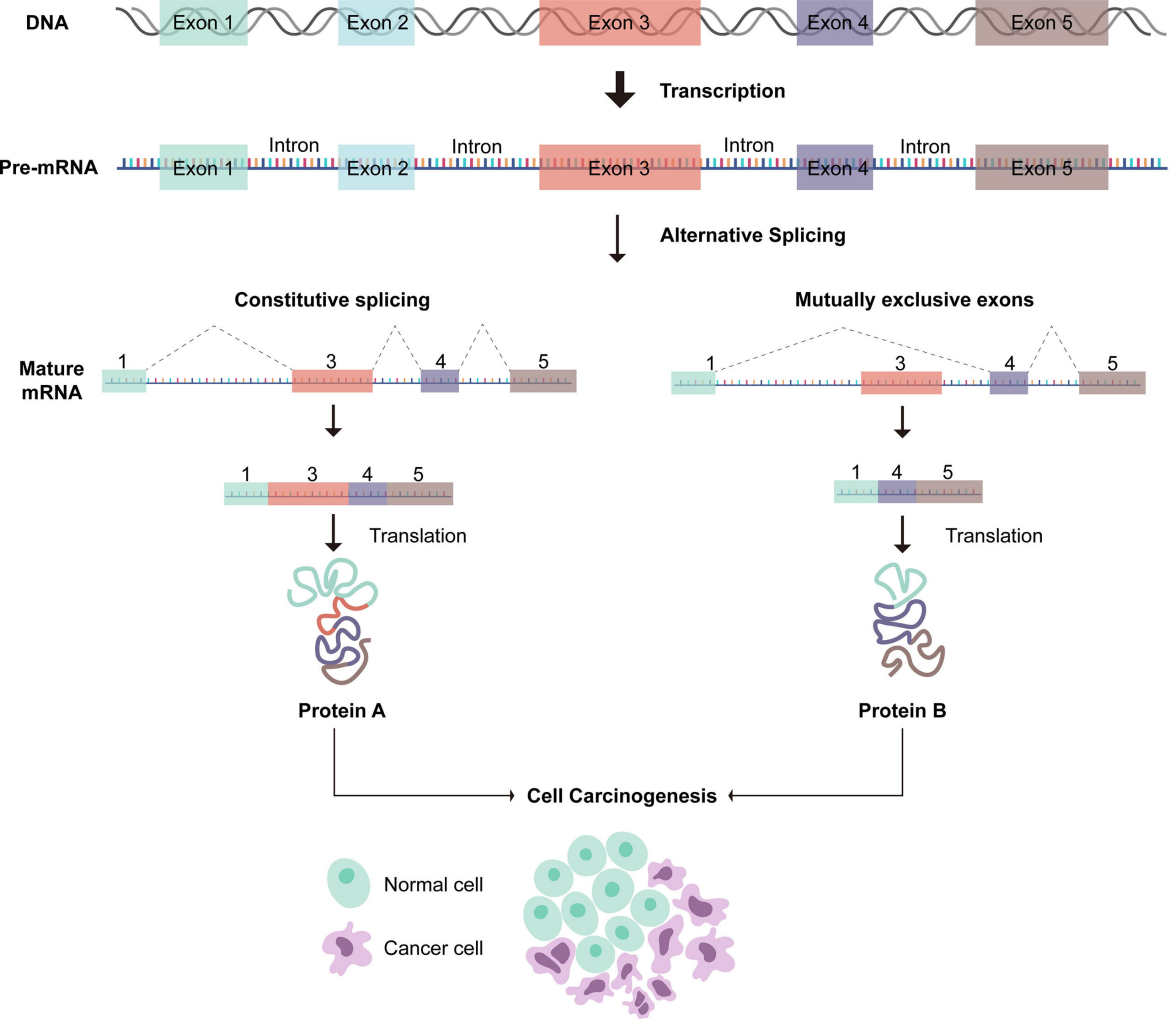
A summary of alterative splicing events.

**Figure 2:**
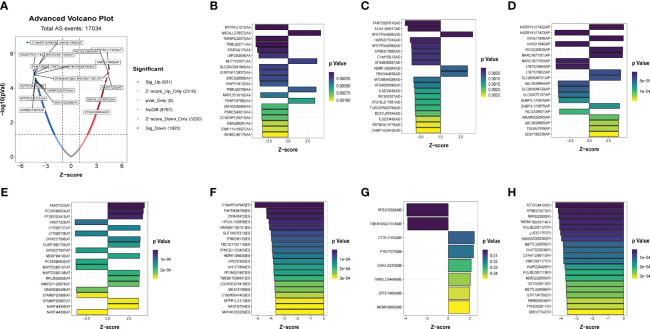
Identification of the prognostic related AS events. **(A)**Volcano plots of prognostic related AS events. **(B-H)** The most significant prognostic related APs, MEs, RIs, AAs, ADs, ATs and ESs in TCGA-BLCA cohort.

A summary of AS events is shown in **Figure 1**. 409 patients with bladder cancer were collected from the TCGA dataset, and thirteen patients with inadequate clinical information were excluded from this research. The clinical profiles of these patients were displayed in **Table 1**. The UpSet diagram comprehensively displays the AS event characteristics (**Figure S1A**). The results demonstrated that exon skip was the prevailing splicing type in bladder cancer, while the mutually exclusive exons had the lowest frequency. Then, a total of 2751 AS events were collected as potential prognostic biomarkers *via* performing univariate Cox regression analysis (p < 0.05). A comprehensive description of the 2751 AS events is shown in **Table S5**. The prognosis-related AS events were delineated using the UpSet diagram (**Figure S1B**). The volcano diagram was plotted to describe the AS events, and the quadrangle map summarizes the first 20 remarkable prognostic-related AS events (**Figure 2**). According to the λ value, the thirteen candidates AS events were selected by performing LASSO regression analysis, including C19orf57|47943|ES, ANK3|11845|AP, ANK3|11842|AP, MARCH6|71561|AP, ACTG1|44120|RI, AK9|77203|AT, DYM|45472|ES, PCSK5|86634|AT, MTFR1L|1212|AA, APBB3|73673|RI, TARBP2|22073|AA, MARS|22600|RI, MICALL2|78572|AA. (**Figure S3**). These independent prognostic-related AS events were chosen to construct AS-based risk models by performing multivariate Cox regression algorism, the risk model is calculated as follows:


Risk score=0.93 × PSI ANK3|11845|AP −1.51 × PSI C19orf57|47943|ES −1.07 × ACTG1|44120|RI+1.83 × AK9|77203|AT – 0.5 × DYM|45472|ES + 0.90 × PCSK5|86634|AT + 3.09×MICALL2|78572|AA.

**Table 1:** Baseline data of all bladder cancer patients.

Characteristics	Type	n	proportion (%)
Age	<=65	161	39.4
	>65	248	60.6
Gender	Female	106	25.9
	Male	303	74.1
Grade	High Grade	385	94.1
	Low Grade	21	5.1
	unkown	3	0.8
Stage	Stage I	2	0.5
	Stage II	130	31.8
	Stage Ill	139	34
	Stage IV	136	33.2
	unkown	2	0.5
T Stage	TO Stage	1	0.2
	T1Stage	3	0.8
	T2 Stage	120	29.4
	T3 Stage	194	47.4
	T4 stage	59	14.4
	unkown	32	7.8
M Stage	MO Stage	194	47.4
	M1Stage	11	2.7
	unkown	204	49.9
N Stage	NO Stage	237	57.9
	N1Stage	47	11.5
	N2 Stage	76	18.6
	N3 Stage	8	2
	unkown	41	10

### Confirmation of Prognostic Model

**Figure 3:**
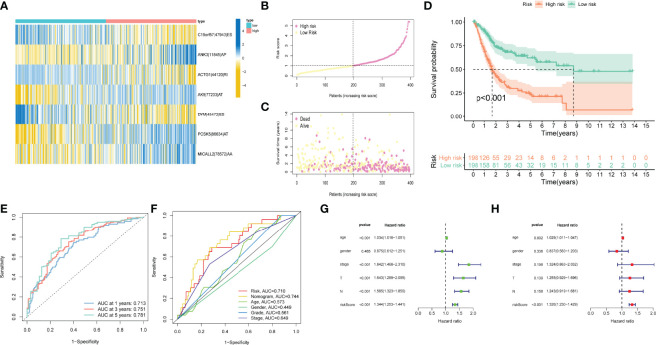
Confirmation of prognostic model. **(A)** Heatmap of the AS events PSI values in bladder cancer. The color from red to green shows a trend from high to low expression. **(B)** The risk score curve exhibits the distribution of prognostic signature risk score. **(C)** The scatter plot exhibits survival times and survival status of bladder cancer patients. **(D)** K–M curve for high- and low-risk groups. **(E)** ROC curves of risk models for overall survival prediction at 1, 3 and 5 years. **(F)** ROC curves for predicting survival with different clinical variables. **(G)** The results of univariate Cox regression analyses. **(H)** The results of multivariate Cox regression analyses.

**Figure 4:**
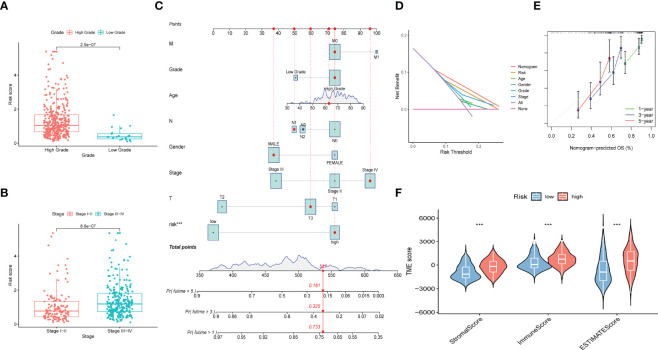
Construction of prognostic nomogram and correlation of immune microenvironment features with different risk scores. **(A)** Risk scores of high and low tumor grades. **(B)** Risk scores of different tumor stages. **(C)** Nomogram established by risk score, age, gender, tumor grade, tumor stage and TNM clinical stage for predicting overall survival probability of bladder cancer patients. **(D)** DCA analysis displayed the prediction performance of selected model. **(E)** Calibration curve of 1‐, 2-, 3-year nomogram, the predicted performances of the model are represented by the 45° gray lines. The green/blue/red line represents 1/3/5 years prediction ability. **(F)** ImmuneScore, StromalScore and ESTIMATE scores of high- and low-risk groups.

Patients were classified into high- and low-risk subgroups for further analysis according to the cut-off value of the median risk score. The level of AS event PSI values in different subgroups is displayed in **Figure 3A**, and the dot plot displays the distribution of patient clinical outcomes (**Figures 3B, C** ). In addition, the K-M analysis indicated that patients with high-risk scores exhibited poor clinical outcomes (**Figure 3D**). The ROC curve was then calculated to evaluate the prognostic value of risk models in bladder cancer patients. The area under curve (AUC) of our risk model at 1, 3- and 5-years was 0.713, 0.751, and 0.781, respectively (**Figure 3E**). The clinical variables and AS-related risk score were consolidated as nomogram model to perform the AUC analysis, we observed that this model gained the highest AUC value (**Figure 3F**), which indicated that the constructed nomogram model had higher sensitivity and specificity for predicting clinical outcomes. In addition, the results of univariate and multivariate Cox regression analyses demonstrated that the risk score can serve as an independent index for bladder cancer (**Figures 3G, H**). In addition, we observed remarkable differences in the risk scores between different clinical variables. The risk score increased with the advancement in clinical pathological stage (p < 0.001, **Figure 4B**) and high-grade tumor subtypes (p < 0.001, **Figure 4A**), which revealed that the risk score was positively related to tumor progression.

To comprehensively assess the prognosis of each patient with bladder cancer, the nomogram model which included the risk score, tumor stage, age, gender, and WHO grade was constructed to evaluate the 1-, 3-, and 5-year overall survival probabilities (**Figure 4C**). DCA analysis also demonstrated that the nomogram model showed the best prediction performance for 1-year OS in bladder cancer (**Figure 4D**). The calibration curve was close to 45°, indicating that the predicted values are close to the predicted values (**Figure 4E**).

### Regulation of AS Events in TIME Alteration

**Figure 5:**
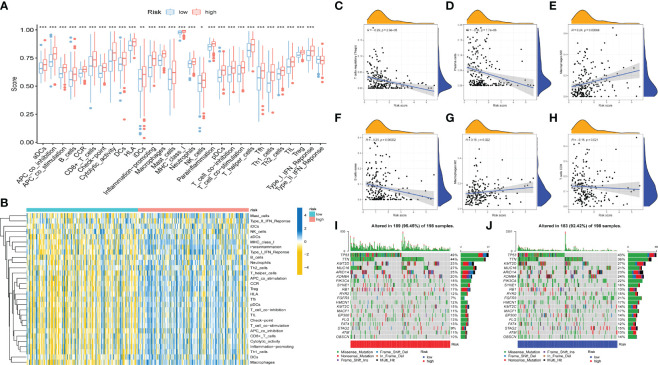
Correlation between AS events and tumor immune microenvironment features. **(A)** Distinction of the immune related profiles between high- and low-risk score groups. The asterisks represented the statistical p value (*P < 0.05; **P < 0.01; ***P < 0.001). **(B)** Heatmap of immune scores and several immune characteristics of two risk score groups. Red indicates high expression and blue indicates low expression. **(C–H)** Correlation analyses of risk score with different immune cells. **(I–J)** The landscape of TMB of bladder cancer with high **(I)** and low risk score.

To further validate whether AS events act as a factor that participates in the formation of the immune microenvironment, “ESTIMATE” R package was employed to calculate the immune score of samples, which displayed those patients with high risk score exhibited higher immune score, stromal score, and ESTIMATE score than those with low risk score (**Figure 4F**). Likewise, the ssGSEA results showed the distinction of the immune-related profiles between the two risk models. The results in **Figures 5A, B** presented the corresponding immune scores of immune-related profiles in high- and low-risk groups. The results showed that the infiltration of immunocytes such as Th1 immunocytes, NK cells, macrophages, aDCs, CD8+ T cells, and neutrophils was remarkably increased in the high-risk group. Immune signatures such as APC co-stimulation, HLA, MHC-class I, and T cell co-stimulation were also increased in the high-risk group. In addition, the CIBERSORT algorithm results revealed that the proportion of T cell regulators, plasma cells, CD8+ T cells, and B cells was negatively correlated with the risk score, and the abundance of resting dendritic cells, macrophages M0, and macrophages M2 were positively associated with the risk score (**Figures 5C–H**). The above results revealed that AS events may play an indispensable role in altering the TIME, and also demonstrated that the constructed risk model may act as a novel biomarker to elaborate the characteristics of immune regulation in bladder cancer.

### Correlation of AS Events With ICB Key Molecules

**Figure 6:**
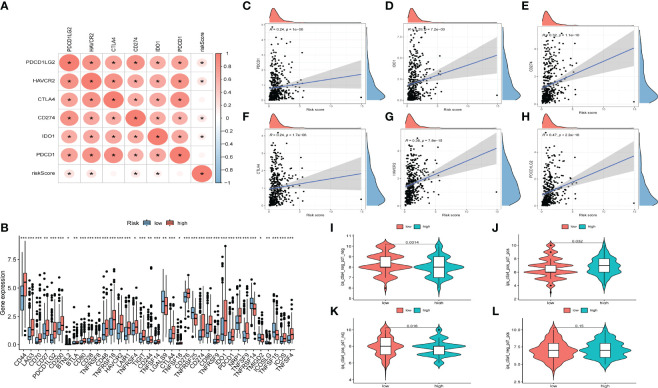
Relationships between AS-based signatures and immune checkpoint blockage key molecules. **(A)** Correlation analyses among six immune checkpoint inhibitors and risk score. **(B)** Difference in expression levels of immune checkpoint blockade-related genes between high- and low-risk groups (*P < 0.05; **P < 0.01; ***P < 0.001). **(C–H)** The positive correlations of CTLA‐4, IDO1, PD‐L1(CD274), TIM‐3(HAVCR2), PD‐L2(PDCD1LG2) and PD‐1(PDCD1) with risk score. **(I–L)** Efficacy score of immunotherapy.

The emergence of immunotherapy has altered the therapeutic landscape of bladder cancer, and the development of immune checkpoint inhibitors broaden the options for clinical decision-making in cancer treatment. First, six ICB key molecules were collected from published articles, including PD‐L1, PD‐1, PD‐L2, TIM‐3, IDO1, and CTLA‐4. Then, correlations between the constructed risk score and ICB key molecules were determined to identify the potential prediction performance of AS events in the immunotherapy of bladder cancer (**Figure 6A**). **Figures 6C–H** shows that the risk score was remarkably positively related to CD274 (R=0.32, *P* =1.1e-10), PDCD1 (R=0.24, *P* =1e-06), CTLA4 (R=0.24, *P* =1.7e-06), HAVCR2 (R=0.38, *P* =7.8e-15), PDCD1LG2 (R=0.47, *P <*2.2e-16), and IDO1 (R=0.29, *P* =7, 2e-09). In addition, we further analysis the association among ICB-related genes and risk score. The results showed that these genes were significantly associated with risk score; LGALS9, TNFRSF25, TNFRSF14, TMIGD2, ICOSLG, and TNFRSF15 were remarkably reduced in patients with high risk scores, while the other genes were significantly up-regulated (**Figure 6B**), suggesting that AS events might serve as a considerable factor in immunotherapy. In addition, we examined the tumor mutation burden between the high (**Figure 5I**) and low risk groups (**Figure 5J**) and found no difference between the two groups. The **Figures 6I-L** displayed the exhibited the effect of immunotherapy between two groups that the effect of immunotherapy in patients with low risk group is more obvious.

### Identification of AS Event-Related Genes

**Figure 7:**
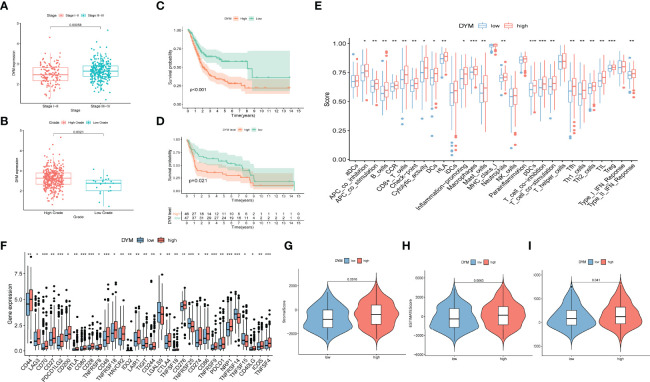
The prognostic significance of DYM in bladder cancer and correlation of DYM with immune checkpoint blockage key genes and tumor immune microenvironment features. **(A)**Significant difference in DYM expression between distinct tumor stages. **(B)** Significant difference in DYM expression between high- and low-grade. **(C, D)** Higher expression level of DYM predicts lower survival probability in TCGA cohort **(C)** and GSE31684 **(D)**. **(E)** Distinction of the immune-related profiles between high- and low-DYM groups (*P < 0.05; **P < 0.01; ***P < 0.001). **(F)** Difference in expression levels of ICB-related genes between high- and low-DYM groups (*P < 0.05; **P < 0.01; ***P < 0.001). **(G-I)** Comparison of ESTIMATE analysis results between high- and low-DYM groups.

There was a total of seven target genes in the constructed risk model. We found that only DYM and MICALL2 genes affected the clinical outcome of bladder cancer. Therefore, the roles of DYM and MICALL2 in bladder cancer were investigated in further analyses. As we found that MICALL2 could not effectively distinguish different clinical pathologies and had little effect on TIME alterations (the results of the MICALL2-related analysis are presented in **Figure S2**), we mainly focused on the *DYM* gene. By investigating the expression level of DYM in bladder cancer tissues with different clinical stages and grades, we observed that DYM was upregulated in bladder cancer tissues with high grade as well as stage III and IV (**Figures 7A, B**). K-M analysis also demonstrated that upregulated DYM was related to poor clinical outcomes in bladder cancer in TCGA database(*P* value <0.001, **Figure 7C**), which was confirmed in GSE31684 (*P* value =0.021, **Figure 7D**). In addition, 32 of the 47 ICB-related gene expression levels were remarkable different between the high and low DYM expression subgroups, and the ICB key molecules (CD274, PDCD1, CTLA4, HAVCR2, PDCD1LG2, IDO1) were upregulated in patients with high DYM expression, suggesting that high expression of DYM might play an important role in mediating immune evasion (**Figure 7F**).

To further demonstrate the relationship between DYM and the immune environment characteristics in bladder cancer, a systematic analysis was conducted as described above. By separating the median DYM expression level, the samples were divided into two subgroups. Outcomes of the “ESTIMATE” analysis revealed that patients with higher DYM expression had a remarkably higher stromal score, immune score, and ESTIMATE score relative to patients with lower DYM expression (**Figures 7G–I**). ssGSEA results showed that the content of infiltration of immunocytes, including Th1 cells, Th2 cells, macrophages, aDCs, CD8+ T cells, NK cells, and neutrophils, and immune signatures such as APC co-stimulation, HLA, MHC-class I, and T cell co-stimulation were remarkably increased in patients with high DYM expression (**Figure 7E**). The above results indicate that the *DYM* gene might be involved in the alteration of the TIME.

**Figure 8:**
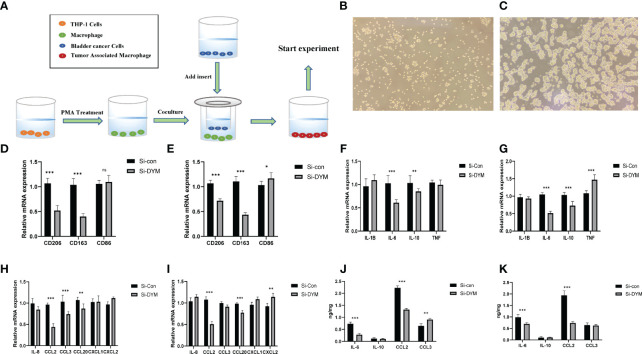
Effects of DYM on tumor immune microenvironment. **(A)** Cell co-culture mechanism diagram. **(B–C)** THP-1 cell **(B)**, M0 macrophage **(C)** photos under microscope. **(D–I)** The expression of macrophage markers **(D, E)**, cytokines **(F–G)** and chemokines **(H, I)** of T24/J82 were detected by QRT-PCR. **(J, K)** ELISA was used to detect the expression of cytokines and chemokine of T24/J82. *P < 0.05, **P < 0.01, ***P < 0.001. ns P > 0.05.

In order to verify the relationship between DYM gene and tumor immune microenvironment, the THP-1 cells were induced to differentiate into M0 macrophages by 100 ng/ml PMA, the **Figure 8B** displayed the THP-1 cell photograph and the **Figure 8C** displayed the M0 macrophages photograph. we co-cultured Si-con or Si-DYM bladder cancer cell lines with M0 macrophages to detect tumor markers of CD206, CD163 and CD86 macrophages (**Figure 8A**). Secondly, we investigated whether DYM gene affected the production of pro-inflammatory factors, immunosuppressive cytokines and chemokines, detected IL-1B, IL-6, IL-10, TNF, IL-8, CCL2, CCL3, CCL20, CXCL1, CXCL2 respectively, and the secretion level was detected with ELISA assay. The results displayed that the levels of CD206 and CD163 were reduced in both two bladder cancer cell lines, while the level of CD86 had no significant difference in T24, and increased in J82 cell line(**Figures 8D, E**). The levels of IL-6, IL-10, CCL2 were significantly decreased in both two bladder cancer cell lines, and Other transcription factors are uncertain in cell lines(**Figures 8F–I**). The ELISA assay exhibited that IL-6, CCL2 were significantly reduced in the supernatant (**Figures 8J, K**).

### Knockdown of DYM Suppressed BCa Cell Proliferation, Invasion, Migration and Promoted Apoptosis *in vitro*

**Figure 9:**
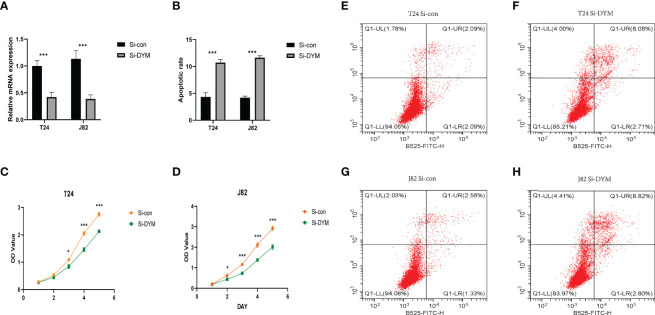
Effects of DYM knockdown on bladder cancer cells proliferation and apoptosis *in vitro*. **(A)** qRT-PCR was used to detect the expression of DYM after T24/J82 cells transfected with DYM siRNA plasmids. **(B, E–H)** Cell apoptosis was detected by flow cytometry after transfection of Si-con or Si-DYM in T24/J82 cells. **(C, D)** The proliferation of T24 **(C)** and J82 cells **(D)** was examined by CCK-8 assay, which exhibited that DYM knockdown group had lower OD value. The representative images are presented. Black lines indicate the wound edge. *P < 0.05, **P < 0.01, ***P < 0.001.

**Figure 10:**
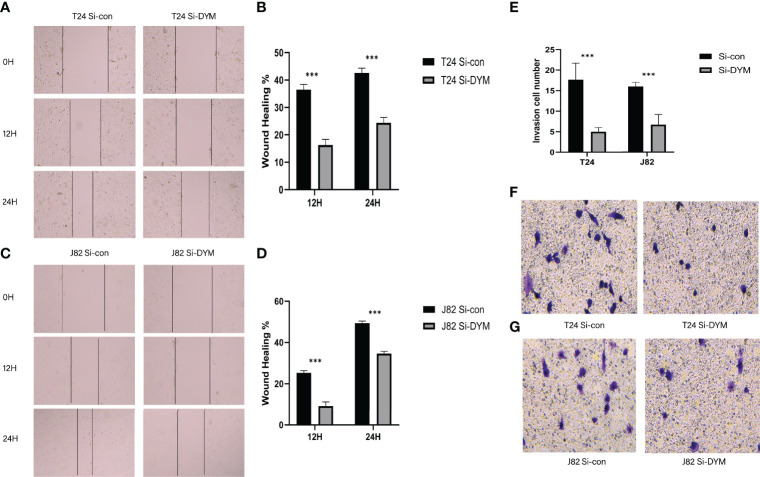
Effects of DYM knockdown on bladder cancer cells invasion and migration *in vitro*. **(A–D)** The wound healing assay was performed to assess the effect of DYM knockdown on the migration of T24 and J82 cells. **(E–G)** Transwell assay was applied to detect the invasion ability of T24 and J82 cells. The representative images are presented. Black lines indicate the wound edge. *P < 0.05, **P < 0.01, ***P < 0.001.

We further examined the biological function of *DYM gene* in the progression of bladder cancer. We designed siRNA targeting DYM, and the knockdown effect of DYM expression level is shown in the **Figure 9A**. The CCK8 assays confirmed that knockdown of DYM remarkably inhibited the proliferation ability of T24 and J82 cells (**Figures 9C, D**). In addition, the flow cytometry analysis displayed the effect of knockdown of DYM genes on cell apoptosis (**Figure 9B**). As shown in **Figures 9E–H**, both early apoptosis and late apoptosis of T24 and J82 cells were increased when transfected with Si-DYM compared with Si-con. Finally, Knockdown of DYM in T24 and J82 cells caused a remarkable reduction in cell invasion and migration ability (**Figures 10A–G**). In summary, our results demonstrated that the *DYM* gene not only plays an immune-related role in bladder cancer, but also promotes the proliferation, migration, metastasis and suppresses cell apoptosis of bladder cancer cells.

## Discussion

Owing to sophisticated molecular mechanisms such as genomic complexity, protein modification diversity, and epigenetics, bladder cancer is highly heterogeneous from a clinical perspective, making it difficult to predict its clinical outcomes accurately (6, 31, 32). Thus, stratifying patients and adopting treatment strategies only *via* pathological anatomy and TNM staging is relatively limited for clinical applications. It is regrettable that the majority of patients with bladder cancer cannot benefit from immunotherapy because of the occurrence of immune escape, mediated by multiple factors (33, 34). Moreover, there are distinct inconsistencies among the responses of patients to both BCG and PD-1/PD-L1 immunotherapy (35, 36). Therefore, the design and development of more reliable prognostic tools for clinical outcome prediction are urgently necessitated, especially for immunotherapeutic prognosis, toward individualized and precise treatment of bladder cancer.

Increasing evidence has elucidated the significance of AS events in physiological or pathological processes, making AS events a novel perspective for understanding intricate pathological processes such as cancer (37, 38). Compared with transcriptome analysis, the investigation based on the alternative splicing level is conducive to the in-depth analysis of the causes, progression and clinical results of diseases. If the up-regulation or down-regulation of genes is caused by alternative splicing alteration, what is the upstream regulatory mechanism and what substances lead to its alteration. Furthermore, the relationship between the alternative splicing modification characteristics of this gene and histone, DNA methylation can be explored. Encouragingly, with the progress in high-throughput sequencing technology, enormous achievements have been made in the study of the latent association between AS patterns and tumors. More importantly, the prognostic ability of AS events has also been widely tested in multiple cancers (14, 39, 40). In bladder cancer, specific AS events have been associated with worse prognosis (41, 42). However, current studies have mainly focused on specific AS events, and systematic analyses of the prognostic value of AS events are minor (43). Fan Z et al. depicted the prognostic signatures of bladder cancer based on AS events *via* comprehensive analysis at the genome-wide level, which revealed that prognostic-related AS events tended to affect the clinical outcome of bladder cancer patients and sensitivity to chemotherapy drugs (44). Nevertheless, a correlation analysis between AS events with TIME and immunotherapeutic outcomes in bladder cancer is still lacking.

In our study, we investigated the AS events in bladder cancer by multiple perspective analysis, selected the most correlative prognostic profiles, constructed a high-preciseness model, and predicted the individual overall survival rates of patients with bladder cancer accurately. Interestingly, the results showed that prognostic predictive signatures established according to the all AS patterns (AD, AA, AP, AT, ES, RI, ME) displayed an appreciable performance for predicting the clinical outcome of bladder cancer patients. Notably, grouped according to clinicopathological factors, these signatures were shown to have excellent prognostic capability. To create an effective and practical tool for clinical practice in bladder cancer, nomogram model contains prognostic characteristics and clinicopathological stages was established, and the predicted results were consistent with the actual results.

With continuous advancement of research into this area, increasing attention has been focused on the crucial role of AS events in TIME (45, 46). Indeed, identifying AS events in TIME might contribute significantly to the bladder cancer treatment. Taking advantage of the ESTIMATE, CIBERSORT, and ssGSEA enrichment analyses, we unveiled the role of AS events in the context of TIME in bladder cancer. The ESTIMATE and ssGSEA results indicated that the group with high-risk scores presented a greater activation of immune and stromal cells. The composition of stromal cells limits the entry of immune cells into the TME to play an anti-tumor role, which explains the poor clinical outcome in the high-risk group with active immune infiltration. The CIBERSORT results agreed with our hypothesis, and found that the risk score was positively correlated with M0 macrophages and M2 macrophages, which favors tumor progression in bladder cancer. Of note, six pivotal ICB targets and 33 ICB-related gene expression levels exhibited a distinct correlation with risk score. In addition, we found the patients with low risk score had superior immunotherapy outcomes, which suggests that risk score might be conducive to developing individual immunotherapeutic strategies and predicting the outcomes (36). Patients with a low risk score may be better candidates for immunotherapy, while patients with a high risk score may prefer chemotherapy or targeted therapy strategies.

DYM encodes a protein that regulates Golgi-associated secretory pathways, which play an indispensable role in the early brain development and endochondral bone formation (47). To date, little is known about the effects of DYM in tumors, especially bladder cancer. This study showed that the high expression of DYM was significantly related to advanced clinicopathology and poor prognosis of bladder cancer. DYM expression was also correlated with TIME alteration and key genes of ICB immunotherapy (e.g., CD274, CTLA4, HAVCR2 and PDCD1). We found that after the knockdown of DYM gene, IL-6, CCL2 cytokines in the supernatant were down-regulated in the co-culture system of bladder cancer cells and macrophages, thereby reducing the recruitment of macrophages(CCL2 effection) and the transformation of macrophages into M2 macrophages(IL-6 effection). There may be a DYM-IL-6 signaling pathway axis. Subsequently, we confirmed that knockdown of DYM genes inhibited the cell proliferation, migration, invasion and promote apoptosis ability of bladder cancer cells.

Overall, subjects with higher DYM expression levels or higher risk scores presented more immune cells in the tumor microenvironment, indicating an enhanced immunophenotype but shorter OS. Consistent with our speculate hypothesize, previous researches have also displayed a correlation between low tumor purity, poor prognosis, and an activated immune phenotype (48, 49). Since the risk scores are related to the expression of ICB-related genes, it can be speculated that the effects of immune cells on tumors may be influenced by ICB pathways. Pan et al. found that bladder cancer with high immune infiltration exhibited a low response rate to ICB therapy, which might support our conjecture (50). These findings suggest that the evaluation of AS events in bladder cancer is conducive to immunotherapeutic choice and prognosis prediction, which has great clinical significance. Presumably, valuable insights into potential therapeutic targets may be revealed by elucidating the mechanisms underlying these events.

Owing to the lack of an ICB treatment dataset related to alternative splicing in the bladder cancer cohort, it was difficult to further explore the association between ICB therapeutic response and risk score. In addition, this study is based on public data sets for bioinformatics analysis, which need be confirmed by our dataset. In the future, we will attempt to collect clinical specimens from bladder cancer patients in Huashan Hospital, obtain alternative splicing event, transcriptome data and clinical information, and conduct prospective validation of this risk model, so as to make this topic more valuable for research. If the effect of DYM gene on tumor immune microenvironment can be verified in immunocompetent mouse model, the topic research in this paper will be further verified. Unfortunately, our laboratory lacks the corresponding experimental technology at present, which is expected to be further improved and optimized in the future.

## Conclusion

Our study provided an indispensable reference for further investigation of the role of AS events in the tumor microenvironment and immunotherapy efficiency, and rendered personalized prognosis monitoring and potential biological treatment targets for bladder cancer.

## Data Availability Statement

All datasets generated for this study are included in the article material, including TCGA database (https://portal.gdc.cancer.gov/), and GEO dataset (https://www.ncbi.nlm.nih.gov/gds/): GSE31684.

## Author Contributions

FY: Conceptualization, Methodology, Writing – review & editing, Investigation. YLia and ZC: Investigation, Project administration. HJ: Conceptualization, Methodology, Writing – review & editing, Supervision. other authors contributed toward data collection and analysis. All authors contributed to the article and approved the submitted version.

## Funding

This study was supported by the National Natural Science Foundation of China (Grant Numbers: 81872102).

## Conflict of Interest

The authors declare that the research was conducted in the absence of any commercial or financial relationships that could be construed as a potential conflict of interest.

## Publisher’s Note

All claims expressed in this article are solely those of the authors and do not necessarily represent those of their affiliated organizations, or those of the publisher, the editors and the reviewers. Any product that may be evaluated in this article, or claim that may be made by its manufacturer, is not guaranteed or endorsed by the publisher.

## Supplementary Material

Supplementary Figure 1
**(A)** Intersections among the seven types of AS events in TCGA BLCA cohort by UpSet diagram. **(B)** Intersections among the seven types of prognostic related AS events by UpSet diagram.

Supplementary Figure 2
**(A)** Tuning parameters (λ/lambda) selected in the LASSO model.

Supplementary Figure 3
**(A)** Kaplan–Meier curve presenting suvival in high and low MICALL2 levels. **(B)** Comparison of MICALL2 expression between high- and low-grade. **(C)** Comparison of MICALL2 expression between major tumor stages. **(D–F)** Comparison of stromal score, immune score and ESTIMATE score between patients with high- and low-MICALL2 groups. **(G)** Distinction of the immune-related profiles between high- and low-MICALL2 groups. **(H)** Difference in expression levels of ICB-related genes between high- and low-MICALL2 groups.
