# Supplementary figures and images for: Immunological Characteristics of Alternative Splicing Profiles Related to Prognosis in Bladder Cancer
Source: Front Immunol. 2022 Jun 13;13:911902. doi: 10.3389/fimmu.2022.911902 (PMC9234272; doi:10.3389/fimmu.2022.911902)

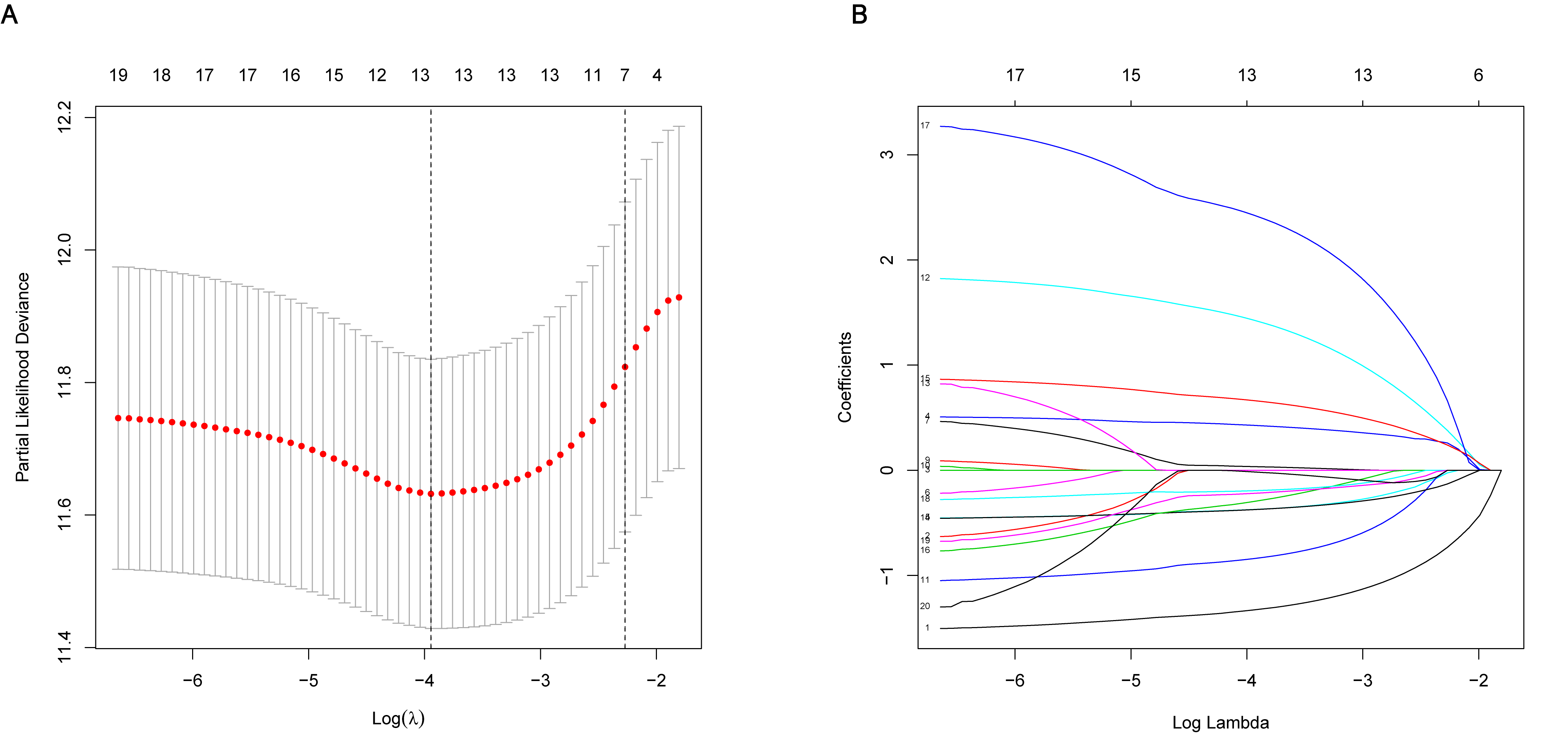

Supplement: Supplementary Figure 1 — (A) Intersections among the seven types of AS events in TCGA BLCA cohort by UpSet diagram. (B) Intersections among the seven types of prognostic related AS events by UpSet diagram. [file Image_1.tif]

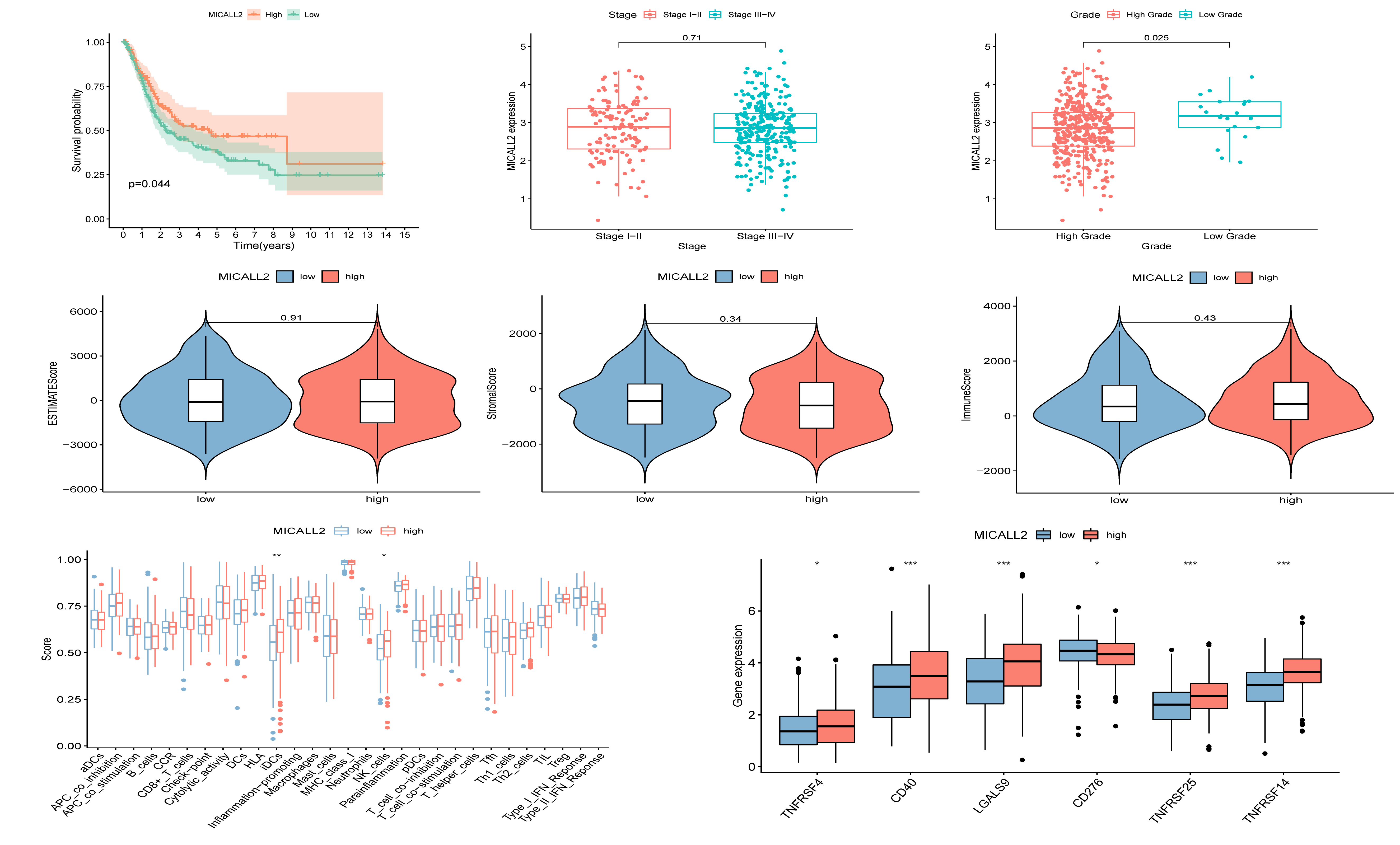

Supplement: Supplementary Figure 2 — (A) Tuning parameters (λ/lambda) selected in the LASSO model. [file Image_2.tif]

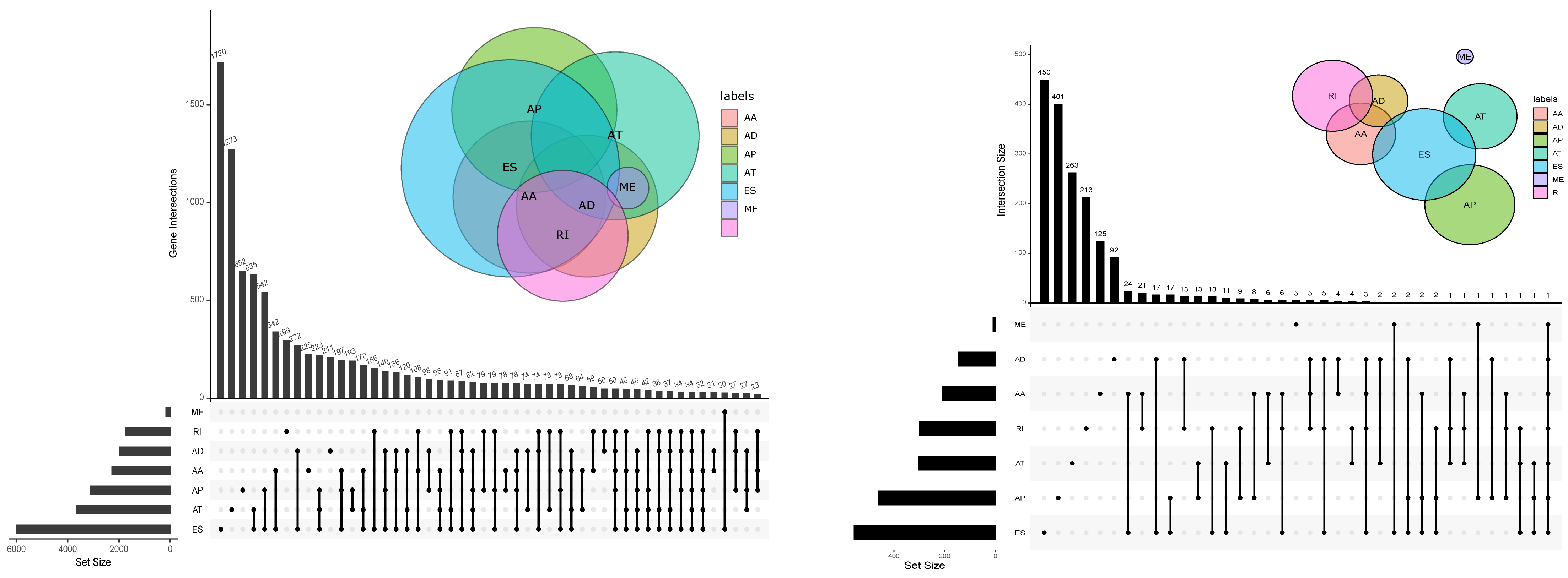

Supplement: Supplementary Figure 3 — (A) Kaplan–Meier curve presenting suvival in high and low MICALL2 levels. (B) Comparison of MICALL2 expression between high- and low-grade. (C) Comparison of MICALL2 expression between major tumor stages. (D–F) Comparison of stromal score, immune score and ESTIMATE score between patients with high- and low-MICALL2 groups. (G) Distinction of the immune-related profiles between high- and low-MICALL2 groups. (H) Difference in expression levels of ICB-related genes between high- and low-MICALL2 groups. [file Image_3.tif]
